# Exogenous abscisic acid and sugar induce a cascade of ripening events associated with anthocyanin accumulation in cultured Pinot Noir grape berries

**DOI:** 10.3389/fpls.2023.1324675

**Published:** 2023-12-21

**Authors:** Jeffrey Bennett, Sathiyamoorthy Meiyalaghan, Han M. Nguyen, Helen Boldingh, Janine Cooney, Caitlin Elborough, Leandro Dias Araujo, Philippa Barrell, Kui Lin-Wang, Blue J. Plunkett, Damian Martin, Richard V. Espley

**Affiliations:** ^1^ Tree Crops, The New Zealand Institute for Plant & Food Research Limited, Motueka, New Zealand; ^2^ Premium Crops & Technology, The New Zealand Institute for Plant & Food Research Limited, Christchurch, New Zealand; ^3^ Tree Crops, The New Zealand Institute for Plant & Food Research Limited, Auckland, New Zealand; ^4^ Fruit Crops Physiology, Biological Chemistry & Bioactives, The New Zealand Institute for Plant & Food Research Limited, Waikato Mail Centre, Hamilton, New Zealand; ^5^ Department of Wine Food & Molecular Biosciences, Lincoln University, Lincoln, New Zealand; ^6^ Viticulture & Oenology, The New Zealand Institute for Plant & Food Research Limited, Blenheim, New Zealand

**Keywords:** sugar, abscisic acid, anthocyanin, grape, Pinot noir, phenylpropanoid pathway, gene expression, ripening

## Abstract

Fruit quality is dependent on various factors including flavour, texture and colour. These factors are determined by the ripening process, either climacteric or non-climacteric. In grape berry, which is non-climacteric, the process is signalled by a complex set of hormone changes. Abscisic acid (ABA) is one of the key hormones involved in ripening, while sugar availability also plays a significant role in certain ripening aspects such as anthocyanin production. To understand the relative influence of hormone and sugar signalling *in situ* can prove problematic due to the physiological and environmental (abiotic and biotic) factors at play in vineyards. Here we report on the use of *in vitro* detached berry culture to investigate the comparative significance of ABA and sugar in the regulation of Pinot noir berry anthocyanin production under controlled conditions. Using a factorial experimental design, pre-véraison berries were cultured on media with various concentrations of sucrose and ABA. After 15 days of *in vitro* culture, the berries were analysed for changes in metabolites, hormones and gene expression. Results illustrated a stimulatory effect of sucrose and ABA on enhancing berry colour and a corresponding increase in anthocyanins. Increased ABA concentration was able to boost anthocyanin production in berries when sucrose supply was low. The sucrose and ABA effects on berry anthocyanins were primarily manifested through the up-regulation of transcription factors and other genes in the phenylpropanoid pathway, while in other parts of the pathway a down-regulation of key proanthocyanindin transcription factors and genes corresponded to sharp reduction in berry proanthocyanidins, irrespective of sucrose supply. Similarly, increased ABA was correlated with a significant reduction in berry malic acid and associated regulatory genes. These findings suggest a predominance of berry ABA over berry sugar in coordinating the physiological and genetic regulation of anthocyanins and proanthocyanins in Pinot noir grape berries.

## 1 Introduction

Fruit quality is partly determined by the success of a ripening signal cascade. The signals that drive the fruit towards maturity are based on genetic, metabolic and hormonal signals, while the overall process can be defined as either climacteric (dependent on ethylene) or non-climacteric (non-dependent on ethylene). Grapes, as a globally significant horticultural crop, serve as an excellent model species for studying non-climacteric ripening. In grape berries, the onset of ripening, known as véraison, is marked by significant changes in hormones, particularly an increase in abscisic acid (ABA) concentration which plays a pivotal role in driving the ripening process (Wheeler et al., 2009; Ferrara et al., 2015; Koyama et al., 2018).

A review of grape berry ripening by Kuhn et al. (2013) highlighted several difficulties that limited progress in understanding grape berry ripening. These included; the lack of easy tools for functional genomics, the high degree of heterogeneity of berry ripening in the bunches, and the lack of reliable and innovative experimental systems to control and study the effects of nutrients and hormones on ripening. The study of grape berry development and ripening *in situ* (vineyard) can also be problematic because of numerous physiological by environment interactions of abiotic and biotic factors such as light, temperature, photosynthesis and carbohydrate partitioning (Kuhn et al., 2013) at the berry, bunch and whole vine level. Since then, improved technologies, including RNA-seq, have shed new light on the cascade of transcriptome changes marking the onset of grape berry ripening and changes thereafter (Fasoli et al., 2018; Tornielli et al., 2023). A system to test the impact of these factors in a controlled environment, without unforeseen variation and influences, would be a useful tool to understand berry specific drivers of ripening and subsequent berry quality. One such system is to utilise *in vitro* berry culture – a form of detached berry tissue culture using berries at predetermined developmental stages. In such a system specific factors or inputs can be individually controlled, for example, temperature or sugar concentration in culture media, to a degree beyond that possible in the heterogenous vineyard situation. The first report of grape berry *in vitro* culture used small (1–2 mm diameter) *Vitis vinifera* L. Muscat of Alexandria berries harvested shortly after fruit set (Bravdo et al., 1990).

Under field conditions grape berry ripening is characterised by host of changes including the rapid accumulation of sugar (Coombe, 1976). The grape berry relies on sucrose translocated from the leaves to the berries to enable this to occur (Mullins et al., 1992). In the berry, sucrose is broken down into glucose and fructose by the action of invertase activity and it is these hexoses that account for the increase in sugar concentration over time (Düring and Alleweldt, 1984). Using an *in vitro* culture system Perez et al. (2000) tested the preference of detached immature berries of *Vitis vinifera* Sultana (5 mm diameter) for their uptake and use of sugar type and contrasted this with field-grown berries. Although cultured Sultana berries did grow and accumulate sugar, they were significantly smaller and lower in sugar concentration than field berries. Results illustrated that berry growth, hexose accumulation and invertase activity increased as sucrose concentration increased up to 15% in the culture medium.

In a similar experiment, Dai et al. (2014) also tested sugar concentration and form, on the ability of pre-véraison Cabernet Sauvignon berries to accumulate sugar and produce anthocyanins after 60–90 days of *in vitro* culture. Results showed that for a given duration of culture, higher sugar (sucrose, glucose or fructose) concentration in the culture medium (up to 16%) resulted in higher berry sugar concentration, faster rate of véraison (colour change), and increased anthocyanin concentrations (mostly peonidin- and malvidin-derived anthocyanins). However, the extent of the increase in anthocyanin was sugar type dependent, with fructose and glucose being more effective in enhancing anthocyanin accumulation in berries than sucrose. Transcriptome analysis of the cultured berries using microarray analysis revealed significant differential gene expression between berries cultured at different sugar concentrations (Dai et al., 2014). Of particular interest in this study was down-regulation of a putative phenylalanine ammonia lyase (PAL), three homologues of flavonoid 3′-hydroxylase (F3′H), a putative flavonoid 3′5′-hydroxylase (F3′5′H), and a putative anthocyanin acyltransferase (ACT). In contrast, putative UDP-glucose:anthocyanidin 3-O-glucosyltransferase (UFGT) gene was up-regulated, while the other structural genes involved in the anthocyanin pathway, including cinnamate-4-hydroxylase (C4H), 4-coumarate-CoA ligase (4CL), chalcone synthase (CHS), chalcone isomerase (CHI), dihydroflavanol 4-reductase (DFR), leucoanthocyanidin dioxygenase/anthocyanidin synthase (LDOX/ANS) and o-methyltransferase (OMT), were not changed.

Abscisic acid is a well-known activator of anthocyanin synthesis in both table and wine grapes (Coombe, 1976; Hiratsuka et al., 2001; Gagné et al., 2006; Wheeler et al., 2009; Gambetta et al., 2010; Ferrara et al., 2015; Koyama et al., 2018). Hiratsuka et al., 2001 showed that 1 g/L ABA added to *in vitro* culture solution induced 2.5 times more anthocyanin pigmentation in *Vitis labruscana* Olympia grape berry halves after 5 days of culture than control (water). A similar study by Deis et al. (2011) found that addition of ABA (up to 1 g/L) to incubated Cabernet Sauvignon berry halves or excised pulp harvested from the berry at 23 or 48 days post véraison increased the concentration of anthocyanins within as little as 4 days after application. Further study of ABA effects on Cabernet Sauvignon berry ripening, with reference to berry skin gene transcriptional activity was reported by Koyama et al. (2010). The study compared exogenous application of ABA to grape bunches on the vine with berries cultured *in vitro* where 750 mg/L ABA was added to the culture medium. The expression of several structural genes in the phenylpropanoid -flavonoid pathways, their transcriptional regulators, as well as genes considered to be involved in the acylation and transport of anthocyanin into the vacuole, were up-regulated by ABA treatment. For example, phenylalanine ammonia lyase (PAL), chalcone synthase (CHS), flavanone-3-hydroxylase (F3′H) and MYB-related transcription factors VvMYBA1&2 and VvMTBPA1 were up-regulated at 14 days after véraison in field berries and at 3 days in cultured berries.

These studies illustrate the individual influence of sugar and ABA on inducing berry ripening including sugar accumulation, anthocyanin synthesis and the correlative link to phenylpropanoid-flavonoid pathway gene expression in the grape berry. However, there are few reports on the combined effect of both sugar and ABA on grape ripening and anthocyanin accumulation. One study by Gambetta et al. (2010) illustrated cultured Cabernet Sauvignon grape berries treated with 10% sucrose and various ABA concentrations began to ripen and change colour while those treated with 2 or 10% sucrose alone remained green; however quantitative measures of berry sugar, ABA and anthocyanins were not undertaken. Here we report on a replicated experiment that investigated the effect of sucrose and ABA on grape berry ripening using an *in vitro* culture system. Using a range of sucrose and ABA concentrations in culture media, we illustrate the complex and nuanced effects of sucrose and ABA on the ripening of Pinot noir berries, including physiological changes in sugars, organic acids, ABA, anthocyanins and other phenolics along with the associated changes in gene expression.

## 2 Materials and methods

### 2.1 Grape berry materials

Grape berries were obtained from field-grown grapevines of *Vitis vinifera* L. cv. Pinot noir (clone UDC5 and rootstock Couderc 3309) planted at Lincoln University, New Zealand. Grapevines were 5 years old, spur-pruned, with a density of 2.4 m between rows and 0.9 m between plants. In midsummer a population of grape bunches exhibiting good fruit set and sound berries was harvested from seven Pinot noir UDC5 grapevines. The timing of the bunch harvest were based on a pre-véraison soluble solids content of 5–6°Brix, measured by ATC portable refractometer BRM-100 (PPS Industries Ltd, NZ). The grape bunches were pooled together before pre-culture sterilisation.

### 2.2 Pre-culture sterilisation

Berries were excised from the harvested grape bunches as small clusters each with two to three berries and while still attached to their rachis. These berry clusters were then placed in a 250-mL plastic pottle (Alto Packaging Ltd, NZ) for batch washing. Each batch contained approximately 90 berries which were gently swirled for 30 s in water containing a drop of antibacterial hand soap liquid. After washing the berries were rinsed and immersed in 70% ethanol for 30 s, and transferred to 0.5% (w/v) sodium dichloroisocyanurate (10 anti-bacterial tablets per litre, Milton^®^ Australia) containing two to three drops of Tween^®^ 20 detergent for 25 min with continuous gentle agitation with the orbital movement of the rotator (PS-M3D multifunction rotator, Grant Instruments Ltd), followed by three rinses with sterile distilled water in a laminar flow hood.

### 2.3 *In-vitro* culture set-up

**Figure 1:**
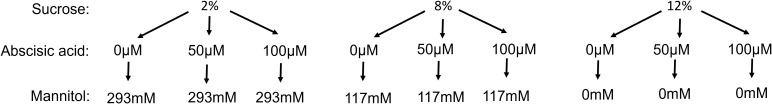
The factorial combination of three sucrose and three abscisic acid concentration treatments and the amount of mannitol required to osmotically balance culture media relative to 12% sucrose.

Tissue culture media chemicals were sourced from Duchefa (M0222, Haarlem, The Netherlands). The tissue culture solid medium composed of half-strength macro-micro elements and vitamins (Murashige and Skoog, 1962) supplemented with varying constituents such as sucrose (bench grade), (±)-Abscisic acid (A1049, Sigma-Aldrich, https://www.sigmaaldrich.com/NZ/en/product/sigma/a1049), and mannitol (M0803, Duchefa) (**Figure 1**). To exclude any effect of osmotic potential of macro additions to culture media (i.e. sucrose), the concentration of sucrose was expressed on a molar basis, and non-assimilable mannitol was used to ensure all samples had the same osmotic potential. Therefore, 21.3 g/L and 53.3 g/L mannitol was added to the 8% and 2% sucrose media preparations, respectively. The pH was adjusted to 5.8 using 1 M KOH prior to the addition of 7 g/L phytoagar (P1003, Duchefa). All media were sterilised using an autoclave at 121˚C for 15 min. After autoclaving, 4ml of medium was distributed in 6-well plates (Jet Biofil) using a sterile syringe.

The surface sterilised berries were aseptically dipped in a sterile 20 mM EDTA solution, and pedicels were cut to approximately 3 mm in the same EDTA solution. Before placing the berries in their respective 6-well culture plates, 150 μL of 5% plant preservative mixture (PPM™, Plant Cell Technology, Inc.), which is a tissue culture biocide, was pipetted onto the surface of the culture medium in each well plate. The solution was carefully swirled around to ensure a protective film formed across the culture medium. Following this step, the berries were placed on the different culture media plates and wrapped with parafilm (PARAFILM^®^ M, Sigma-Aldrich) (**Supplementary Figure 1**). Plates were incubated for 15 days at 24 ± 1°C under cool white fluorescent lamps with a 16‐h photoperiod.

## 3 Experimental methods

Three concentrations of (±)- Abscisic acid, namely 0, 50 and 100 µM were combined with three concentrations of sucrose, specifically 2, 8 and 12%, resulting in a total of nine treatments (**Figure 1**). The experimental design followed a randomised factorial approach, with each treatment being replicated five times using 6-well culture plates with 6 berries as the experimental unit. The osmotic potential of the media containing 2 or 8% sucrose was balanced relative to 12% sucrose by the addition of mannitol as described above. The lower concentrations of ABA were considered to have no influence on the osmotic potential of the media; therefore, additional mannitol was not used.

### 3.1 Sample preparation for analysis

Freshly harvested berries from the experiment were wiped clean to remove any culture media or moisture. The pedicel was removed and berry fresh weight measured before the berries were placed in replicate vials and frozen at -70°C. At the beginning of sample preparation for chemical analysis, the frozen berries were cut in half to remove the seeds. The deseeded berries were then ground to fine powder in a mortar and pestle using liquid nitrogen to keep the sample frozen.

#### 3.1.1 Berry sugar and organic acid analyses

Approximately 200 mg of the frozen homogenised powdered grape tissue was extracted using 5 mL of 80% ethanol with the addition of adonitol (02240, Sigma-Aldrich) as an internal standard and incubated for 1 h at 60°C. The samples were mixed and then centrifuged. A subsample of the supernatant was taken, and dried using a Centrivap (Labogene Scanvac Coolsafe, Denmark). The sample was then re-dissolved in ultrapure water. The soluble sugars (fructose, glucose, mannitol and sucrose) were analysed using a DIONEX ICS-5000 Reagent-Free™ IC (RFIC™) system with a CarboPac PA20 column. Berry organic acid analysis was performed on an aliquot of frozen homogenised powdered grape tissue. Samples (0.1 g) were extracted with cold 0.5% meta-phosphoric acid (0.5 mL) with shaking (1 hr, 4°C). After centrifugation at 3200 rpm (15 min, 4°C), an aliquot of the supernatant was transferred to an autosampler vial for analysis by Ultra High Pressure Liquid Chromatography (UHPLC). Tartaric, citric and malic acid concentrations were measured using a Dionex UltiMate 3000 Series UHPLC (ThermoFisher Scientific, San Jose, CA, USA) with PDA (photodiode array) detection at 210 and 220 nm. The UHPLC analysis included standards for calibration and quantitation, spikes for recovery, and duplicates for reproducibility. The organic acid UHPLC peak areas were integrated and the concentration expressed as mg/g berry fresh weight. Total acid was expressed as sum of the three organic acids analysed; it is not equivalent to titratable acidity used for grape juice and wine.

#### 3.1.2 Berry phytohormone and phenylalanine analyses

Phytohormone concentrations were determined by liquid chromatography mass spectrometry (LC-MS) on a 7500 QTrap triple quadrupole/linear ion trap (QqLIT) mass spectrometer equipped with a TurboIon-Spray™ interface (AB Sciex, ON, Canada) coupled to a Shimadzu Nexera UHPLC (Kyoto, Japan) as previously described (Wurms et al., 2023; Bulley et al., 2021). Aliquots of frozen powdered grape berry material were weighed (100 mg) and to each was added chilled (4°C) extraction solvent (acetonitrile + 0.01% TFA) (1mL), labelled internal standard mix and stainless steel beads 0.9–2 mm (0.8 g) (Next Advance Inc., Raymertown, NY, USA). Samples were bead beaten for 5 min (Bullet Blender 24 Gold, Next Advance Inc., NY, USA) before being extracted overnight at 4°C using an end-over-end rotator at 30 rotations/min. After centrifugation at 16,000× g for 5 min, supernatant from each sample was transferred into a well in a 96-well collection plate (Phenomenex, Torrance, CA, USA). The remaining pellet was re-extracted with the chilled (4°C) extraction solvent (0.5 mL), combined with the first supernatant, and evaporated to dryness using a CentriVap concentrator (Labconco, Kansas City, MO, USA) at −4°C. Sample clean-up employed graphitised carbon following a method described by Cai et al. (2016), with modifications to adapt to a 96-well plate format. Briefly, samples were reconstituted in chilled (4°C) 80:20 acetonitrile:water (1 mL) and shaken for 20 min on a flat-bed orbital shaker before SPE clean-up on a Hypersep Hypercarb 96-well plate (25 mg/1 mL; Thermo Scientific, CA, USA). Plates were conditioned using acetonitrile (1 mL) followed by water (1 mL). After conditioning, samples were loaded and the acidic plant hormones were eluted with acetonitrile (0.5 mL) and evaporated to dryness using a CentriVap concentrator at −4°C. Samples were reconstituted in 10:90 acetonitrile:water (0.2 mL) for analysis by LC-MS. The LC-MS phytohormone analysis methodology was extended to include quantification of phenylalanine by addition of 15 ng of 13C6-L-phenylalanine to the labelled internal standard mix used to spike each sample at extraction. MS transitions monitored in the negative mode were phenylalanine 164>147 and 13C6-L-phenylalanine 170>108.8. Concentrations of hormones and phenylalanine were calculated on the basis of the LC-MS peak area for the endogenous compounds relative to those determined for the internal standards and were expressed in ng/g berry fresh weight.

### 3.1.3 Berry phenolic and anthocyanin analyses

An aliquot (300 mg) of frozen homogenised powdered berries was transferred to a 5-mL centrifuge tube and extracted using a method adapted from Bindon et al. (2014). In brief, grape homogenates were extracted using 2-mL of an acidified ethanolic solution (50% v/v, 0.05 N HCl). The mixture was sonicated for 90 minutes followed by centrifugation (5000 rpm, 5 min) and filtration (0.22 um). Filtered extracts were injected into a Waters Acquity I-Class Plus UPLC (Waters, Milford, MA, USA) with a binary pump, autosampler coupled to a photodiode array detector. The UPLC method used to analyse monomeric phenolics and total polymeric tannins was based on the work of Peng et al. (2002) and Garrido-Bañuelos et al. (2019). Data were processed using Waters Empower 3 build 3471. Catechin and epicatechin were monitored at 280 nm; hydroxycinnamic acids (quantified using caffeic and coumaric acids) at 320 nm; flavonol glycosides (as rutin equivalents) and aglycones (as quercetin equivalents) at 360 nm; and anthocyanins at 520 nm (as malvidin-3-glucoside (M3G) equivalents). Total tannin was quantified as epicatechin equivalents by integrating the broad peak at the end of the run at 280 nm. Concentrations of monomeric phenolics and total polymeric tannins were determined and expressed on a mg/kg berry fresh weight basis and were the average of single UPLC injections of duplicate sample extractions.

All metabolite data, including sugars, organic acids, phytohormones, phenolics and anthocyanins were analysed against the factorial experimental design using ANOVA (Genstat Ver. 22, VSN International Ltd.) for main and interaction effects. Statistical probability values and least significant differences (α = 0.05) were presented in tabled and graphed results.

#### 3.1.4 RNA extraction, RNA-seq and qPCR analysis

A 200–400 ng berry tissue aliquot was used for total RNA isolation using a Spectrum™ RNA extraction kit (Sigma-Aldrich) following the manufacturer’s protocol. RNA was visualised by gel electrophoresis and quantification was performed using NanoDrop™ ND-1000 UV-Vis Spectrophotometer (NanoDrop Technologies, Thermo Scientific, USA). The QuantiTect^®^ Reverse Transcription Kit, with integrated removal of genomic DNA contamination, was used for cDNA synthesis (QIAGEN Group).

mRNA-sequencing including library preparation, sequencing and quantification analysis were performed by Novogene (HK) Company Limited (Hong Kong, China). The transcriptome was sequenced using the Illumina NovaSeq 6000 sequencing system using a 150bp-paired end strategy. Reads are mapped onto the *Vitis vinifera* reference genome (Genome ID ensemblplants_vitis_vinifera_12x_gca_000003745_2) using the HISAT2 software (version 2.0.5) (Kim et al., 2019). Genes with an adjusted *p*-value ≤ 0.05 and |log2FoldChange| ≥ 1.0 were identified as differentially expressed using the EdgeR R package (version 3.22.5) (Robinson et al., 2010). The *p*-values were adjusted using the Benjamini & Hochberg method. KEGG and GO analysis was performed with the clusterProfiler R package (version 3.8.1) (Yu et al., 2012). Terms and pathways with adjusted *p*-values < 0.05 were considered significantly enriched.

Quantitative polymerase chain reaction (qPCR) was performed using the Roche LightCycler^®^ 480 system and LightCycler SYBR Green I Master. Reactions contained 5 µL Master Mix, 0.5 µL of each primer (10 µM), 2.5 µL diluted cDNA (1:20), and nuclease free water to a volume of 10 µL. Analysis was performed based on method described by André et al. (2009).

Two housekeeping genes (control genes with stable transcript abundance), Actin and GAPDH, were included in each experiment and used to normalise expression data. Data shown are the means of four technical replicates. The primers are listed in **Supplementary Table 1**.

## 4 Results

### 4.1 Effect of ABA and sucrose on berry weight and primary metabolism – sugars and organic acid

**Figure 2:**
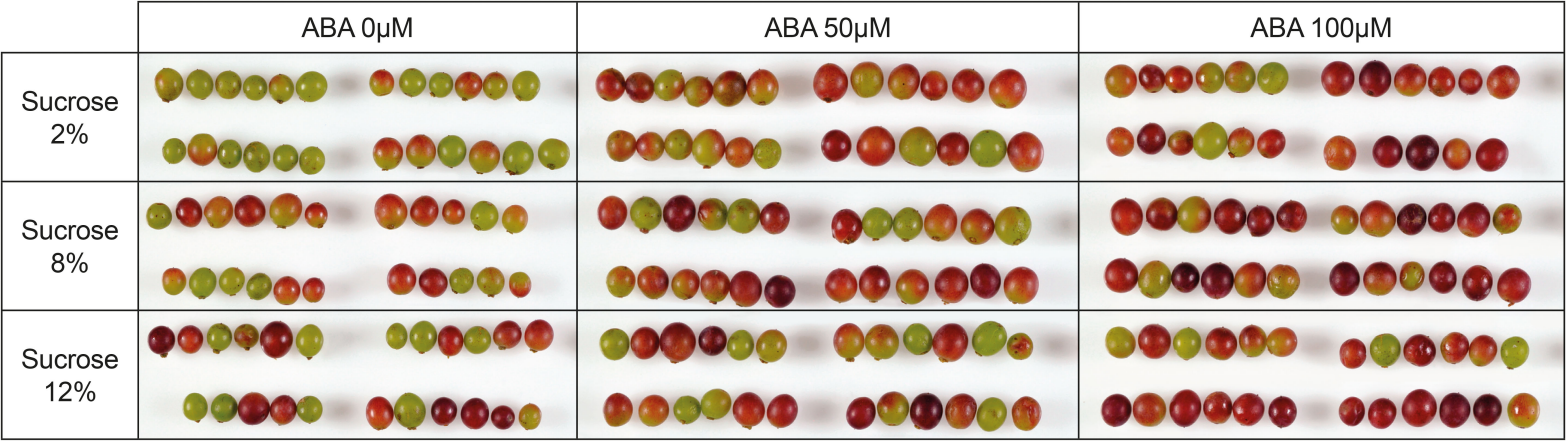
A compiled image showing the effects of culture medium sucrose and abscisic acid (ABA) concentration treatments on the colour of Pinot noir berries after 15 days of *in vitro* culture. All berries were green at the beginning of the experiment (pre-culture). Replicates 1-4 are displayed in the image.

During the experimental period many berries changed from green (pre-culture) (**Supplementary Figure 1**) to being partially or fully coloured (**Figure 2**). In the absence of ABA more berries were pigmented at 8 and 12% sucrose than at 2% sucrose. The ABA treatments of 50 and 100 µM also resulted in more highly coloured berries than no ABA treatment, regardless of sugar concentration (**Figure 2**).

Berry fresh weight was not affected by sucrose or ABA treatments, but increased by 7% after 15 days’ culture compared to pre-culture (**Table 1**). Likewise, berry sugar content increased by 31% after 15 days of culture. In the absence of ABA, berry fructose concentration was increased by 8 and 12% sucrose, and also significantly increased by ABA but only at 2% sucrose (**Table 1**). However, changes in fructose concentration did not result in significant differences in berry sugar content between treatments (**Table 1**). Some mannitol was detected in berries cultured on 2 and 8% sucrose, an artefact of the osmotic balancing of media using mannitol. Organic acid results showed that total acid concentration reduced by 18% after 15 days of culture compared with pre-culture (**Table 1**). Both 50 and 100 µM ABA treatment significantly reduced total acid concentrations at 2 and 8% sucrose but had little influence at 12% sucrose. In both cases, the reduction in total acid over the course of the experiment and in response to ABA treatment was attributed to a reduction in malic acid concentration.

**Table 1:** The effects of medium sucrose and abscisic acid (ABA) concentration treatments on Pinot noir berry parameters and biochemical composition including sugars and organic acids after 15 days of *in vitro* culture.

Sucrose concentration		2%			8%			12%			Statistical significance			
ABA concentration (µM)	Pre-culture	0	50	100	0	50	100	0	50	100	*p*-value Sucrose	*p*-value ABA	*p*-value Interaction	LSD (α = 5%)
Berry parameters														
Mean berry weight (g)	0.60	0.69	0.61	0.70	0.62	0.64	0.66	0.65	0.64	0.57	0.349	0.735	0.331	0.12
Berry sugars (concentration - mg/g fresh weight)														
Mannitol	nd*	2.6	2.5	2.3	0.8	0.7	0.8	nd	nd	nd	<.001	0.486	0.259	0.38
Glucose	15.3	15.8	18.6	19.4	18.1	21.9	21.0	19.6	19.7	18.2	0.290	0.316	0.648	5.317
Fructose	7.4	6.6	10.3	11.0	9.5	12.2	12.3	11.1	11.3	10.5	0.036	0.014	0.151	2.836
Sucrose	0.8	0.5	0.6	0.7	1.0	0.9	0.9	1.1	0.9	0.9	<.001	0.697	0.213	0.257
Total sugar	24.0	23.3	29.9	31.4	29.0	35.3	34.5	32.3	32.4	30.0	0.115	0.131	0.412	8.02
Berry sugar content**	13.6	14.3	16.0	20.9	16.4	20.5	21.9	17.5	19.2	15.4	0.340	0.197	0.256	6.57
Berry organic acids (concentration- mg/g fresh weight)														
Malic acid	19.7	16.8	13.0	12.4	15.0	13.1	12.2	14.3	14.2	13.4	0.397	<.001	0.040	1.79
Tartaric acid	9.5	9.7	10.1	9.7	10.0	9.8	10.1	10.0	10.0	10.1	0.465	0.871	0.604	0.65
Total acid	29.5	26.8	23.4	22.3	25.3	23.2	22.5	24.6	24.6	23.8	0.495	<.001	0.086	1.968

pre-culture is not statistically compared with sucrose and ABA treatments. *nd, not detected. **Calculated amount of sugar per berry (mg). p-values ≤ 0.05 are considered statistically significant.

### 4.2 Effect of ABA and sugar on secondary metabolism – phytohormones and phenolics

**Figure 3:**
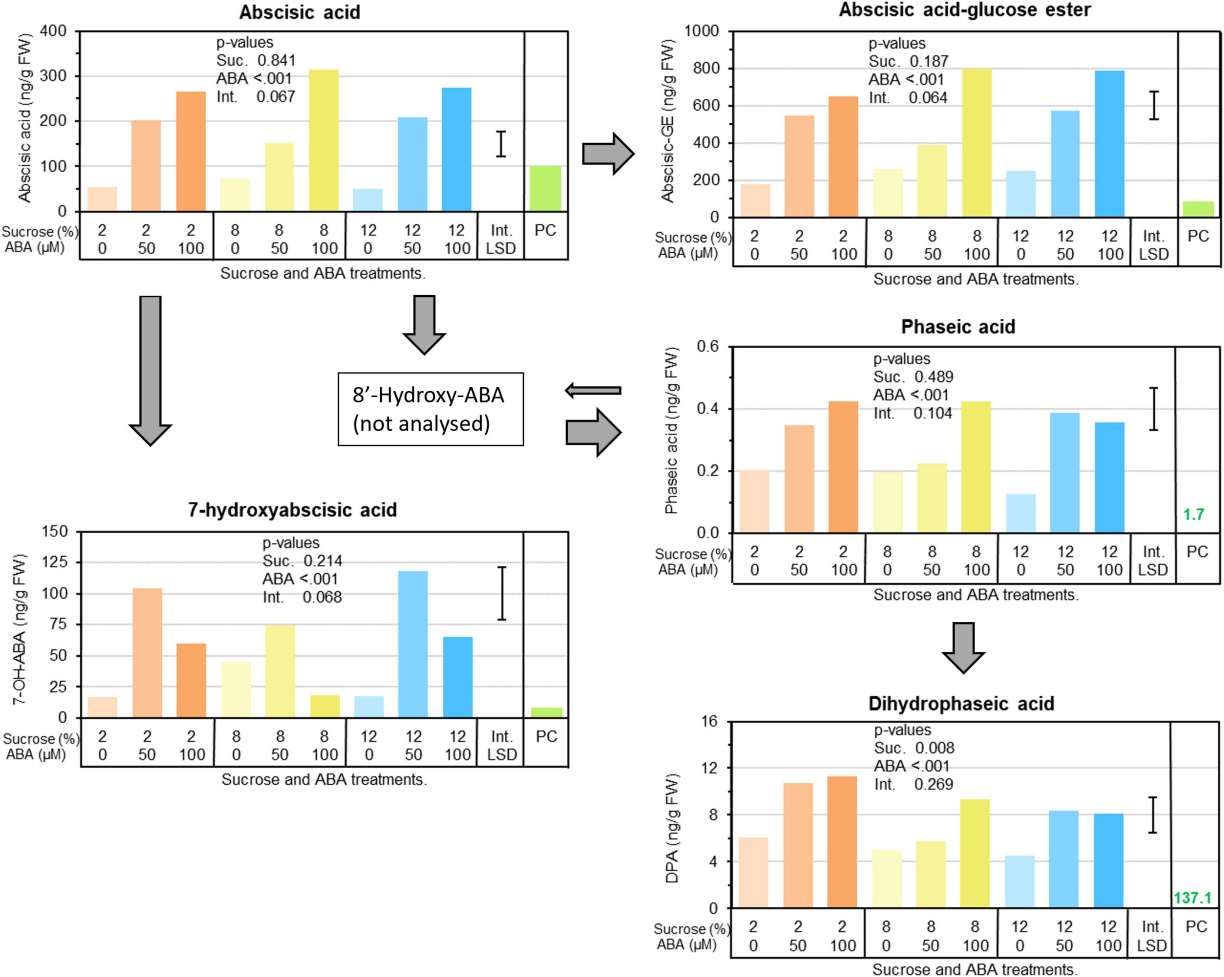
The effects of culture medium sucrose and abscisic acid (ABA) concentration treatments on Pinot noir berry abscisic acid metabolism, including berry concentrations of free ABA, storage ABA (ABA-glucose ester) and various ABA metabolites (7-hydroxyabscisic acid, phaseic and dihydrophaseic acid) after 15 days of *in vitro* culture. Note pre-culture (PC) is not statistically compared with sucrose and ABA treatments. P-values for main and interaction effects are presented in each graph. Error bar represents interaction LSD (α = 5%).

Berry ABA concentrations significantly increased in response to ABA treatment, elevating them well above those found in pre-culture berries and in non-ABA treated berries (up to 380% higher) (**Figure 3**). Similarly, the storage form (abscisic acid-glucose ester; ABA-GE), and downstream metabolites, phaseic acid and dihydrophaseic acid, were also increased in response to ABA treatment (**Figure 3**). Abscisic acid-glucose ester was up to 225% higher. Regardless of ABA treatment, approximately 3 times more ABA-GE was present in berries than free (**Figure 3**). Sucrose treatment had little effect on ABA metabolism.

**Figure 4:**
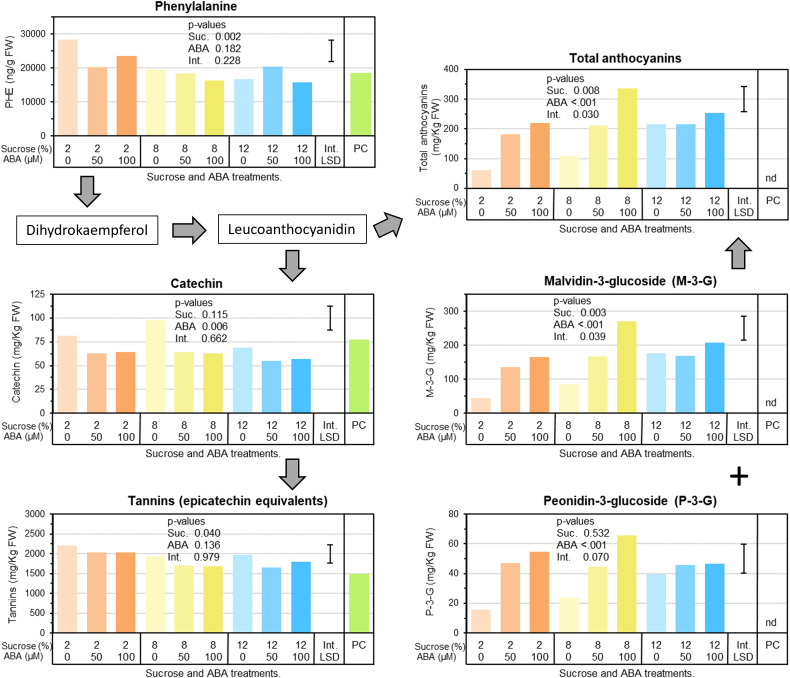
The effects of culture medium sucrose and abscisic acid (ABA) concentration treatments on Pinot noir berry phenylpropanoid pathway metabolism, including berry concentrations of phenylalanine, phenolic acids, flavan-3-ols and anthocyanins after 15 days of *in vitro* culture. Note pre-culture (PC) is not statistically compared with sucrose and ABA treatments. P-values for main and interaction effects are presented in each graph. Error bars represents interaction LSD (α = 5%). nd = not detected.

Berry phenylalanine concentrations were significantly reduced by 22% in berries cultured at 8 and 12% sucrose compared with berries cultured at 2% sucrose, while ABA treatment had no significant effect and there was no interaction between treatments (**Figure 4**). In terms of phenolic acids, caftaric and coutaric acid were significantly reduced by both ABA and sucrose treatments. For ABA the effect was dosage independent, while 8% sucrose reduced concentrations the most. In contrast, benzoic acid showed a small but significant increase in concentration in response to ABA treatment. Berry quercetin glycoside concentrations were not affected by ABA or sucrose treatment (**Supplementary Figure 2**). Berry catechin concentrations were reduced by ABA treatments, but not by sucrose. On average, both ABA concentrations (50 or 100µM) reduced catechin by 26% compared with no ABA treatment (**Figure 4**). In contrast, tannin was reduced by sucrose, but not altered by ABA treatment. On average, either 8 or 12% sucrose reduced tannin by 14% compared with 2% sucrose. Two forms of anthocyanin were detected in berries: malvidin-3‐glucoside (M-3-G) and peonidn-3-glucoside (P-3-G) and both were significantly increased by ABA treatment (**Figure 4**). The effect appeared to be dosage related, with the 100µM ABA treatment increasing anthocyanin concentration more than the 50µM ABA treatment. For M‐3‐G, the most abundant anthocyanin, there was a significant sucrose by ABA treatment interaction whereby with 2 or 8% sucrose, increasing ABA treatment dosage from 50 to 100µM increased M‐3‐G concentration, but with 12% sucrose, high concentrations of M-3-G were present regardless of ABA dosage treatment. Total anthocyanins (sum of M-3-G and P-3-G) reflected the M-3-G results (**Figure 4**).

### 4.3 Influence of ABA and sucrose on the RNA transcriptome of the berry using RNA-seq and qPCR analyses

**Figure 5:**
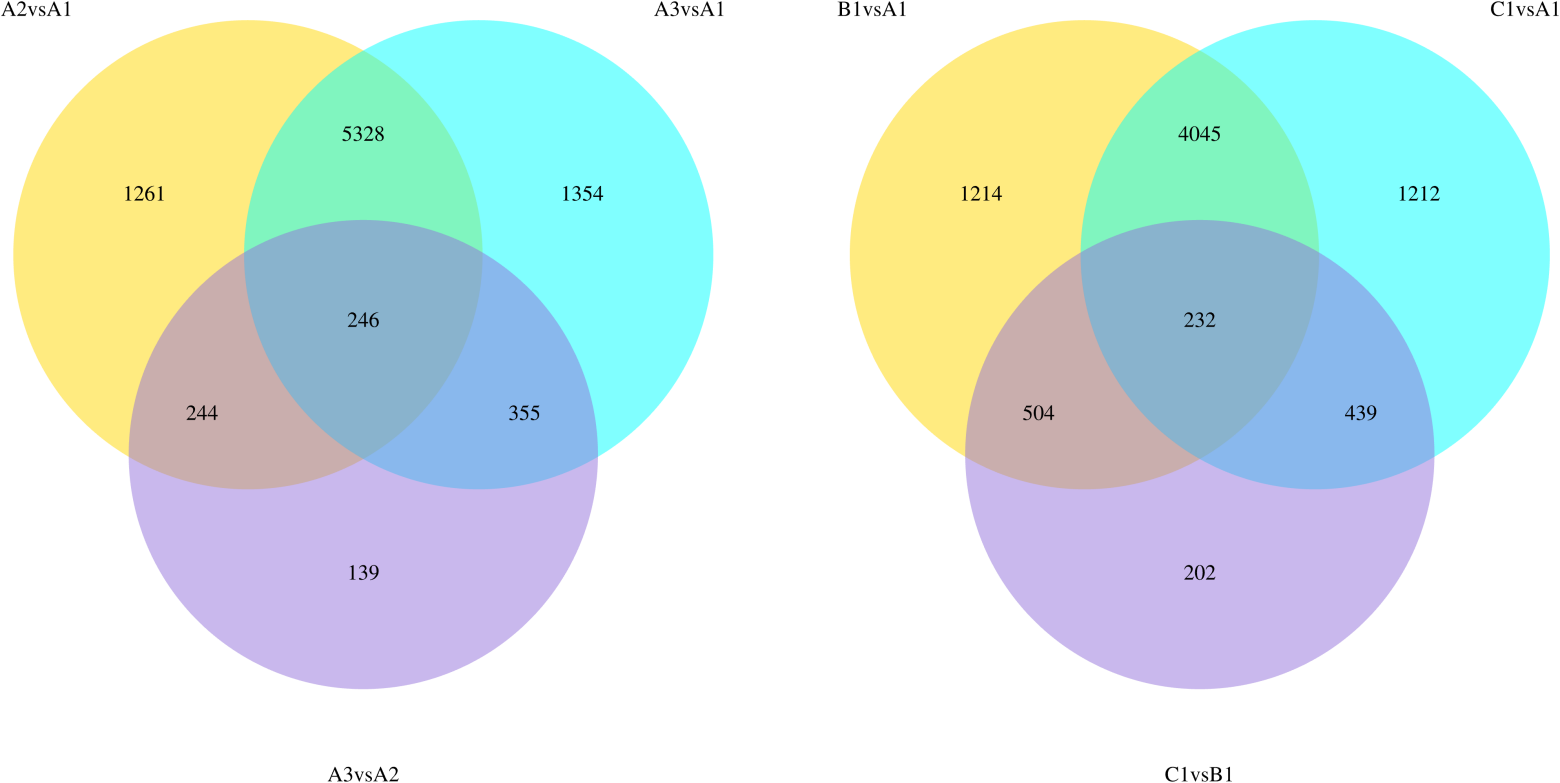
Venn diagram showing differentially expressed genes between treatments. A1 – 2% sucrose, 0μM ABA. A2 – 2% sucrose, 50μM ABA, A3 – 2% sucrose, 100μM ABA, B1 – 8% sucrose, 0μM ABA, C1 – 12% sucrose 0μM ABA.

The introduction of ABA or high sucrose into the berry culture media induced considerable changes in the transcriptome. At 2% sucrose, a total of 7079 and 7283 genes were differentially expressed when ABA concentration was at 50μM and 100μM respectively compared to 0μM ABA (**Figure 5**). Similarly, at 0μM ABA, when sucrose concentration was at 8% and 12%, there were 5995 and 5928 differentially expressed genes respectively, compared to 2% sucrose (**Figure 5**). However, when either ABA concentration or sucrose concentration were already elevated, the transcriptome was less affected by changes in ABA or sucrose concentration. For example, at 2% sucrose, high ABA concentration resulted in 984 differentially expressed genes. Similarly, at 0% ABA, high sucrose resulted in 1377 differentially expressed genes (**Figure 5**). KEGG and GO analyses were performed, showing enriched terms and pathways for sucrose and ABA treatments: ‘phenylpropanoid metabolism’, ‘phenylpropanoid catabolism’, ‘flavonoid biosynthesis’, ‘phenylpropanoid biosynthesis’, ‘plant hormone signal transduction’, ‘stilbenoid, diarylheptanoid and gingerol biosynthesis’ (**Supplementary Figure 5**).

**Figure 6:**
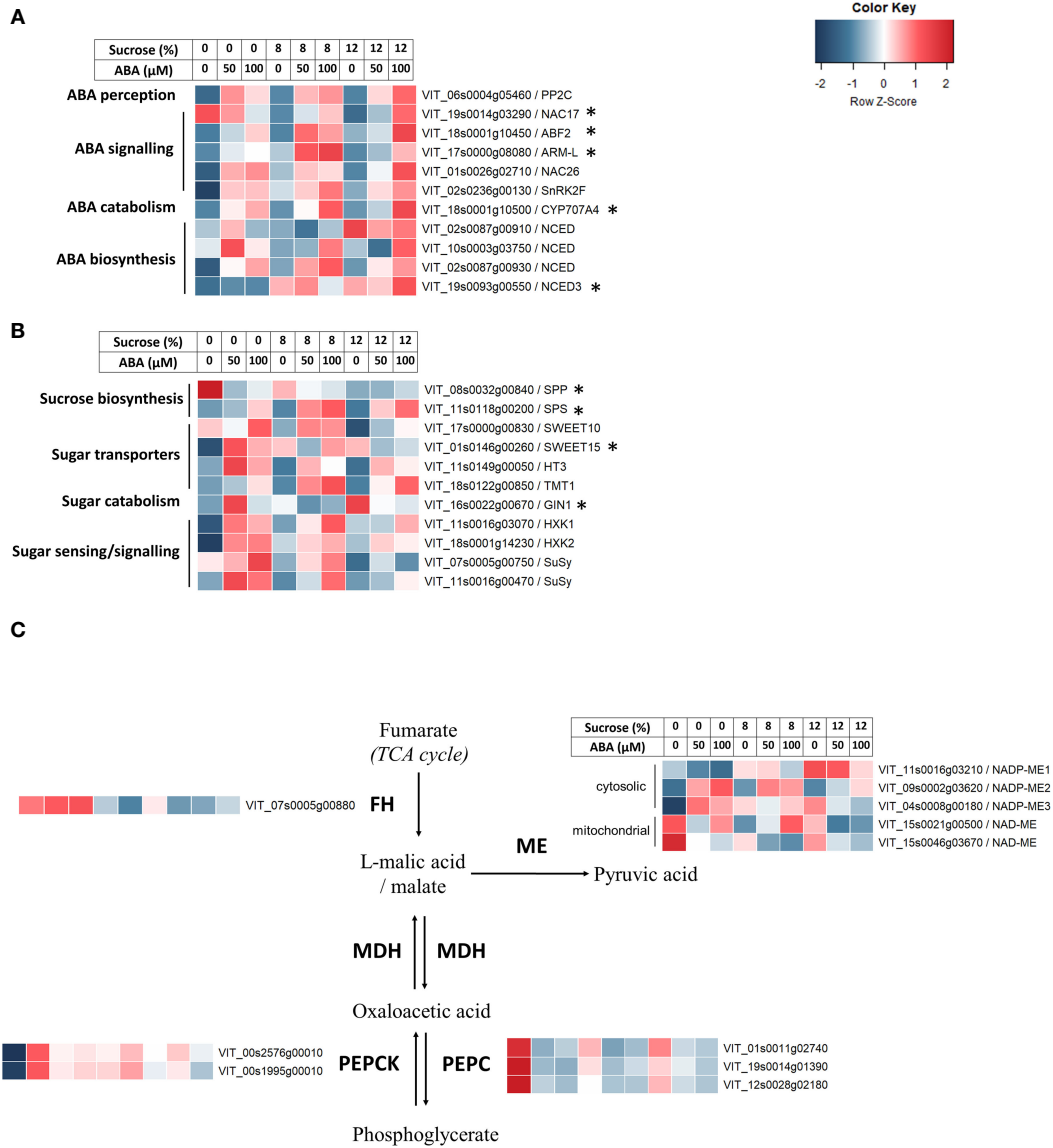
The effect of culture medium sucrose and abscisic acid (ABA) concentration treatment on RNA-seq derived expression of Pinot Noir berry genes. **(A)** ABA-related genes. **(B)** Sugar-related genes. **(C)** Malic acid-related genes. Data were Z-score standardised to -2 to +2 for each gene. Genes with FPKM count <2 for all treatments were excluded. *Gene expression also determined by quantitative polymerase chain reaction (qPCR).

In response to the increase in culture medium ABA concentration, the heat maps of RNA-seq analysis showed a clear increase in the expression of genes involved in ABA perception (e.g. *VvPP2C*), signalling (e.g. *VvSnRK2F*, *VvNAC26*, *VvARM-L*), biosynthesis (e.g. *VvNCED*, VIT_02s0087g00930) and catabolism (e.g. *VvCYP7A4*) were observed (**Figure 6A**). Additionally, an NCED isoform (*VvNCED3*, VIT_19s0093g00550) was up-regulated with higher sucrose concentration rather than ABA. The q-PCR analysis indicated the ABA catabolism gene *CYP7A4* was strongly up-regulated in stepwise fashion (**Supplementary Figure 3**), and this mirrored the production of the downstream products (phaseic and dihydrophaseic acids) of its substrate 8-hydroxyabscisic acid (**Figure 5**). The ABA-responsive genes ABF2, ARM-L and NAC7, which are involved in ABA signal transduction, were generally less responsive to sucrose and ABA treatments.

Transcriptional responses to changes in medium composition were also observed in sugar-related genes. The expression of the bidirectional sugar transporter *VvSWEET15* and the hexokinases *VvHXK1/2* were up-regulated following both increased ABA and sucrose concentration (**Figure 6B**). One Sucrose Synthase (*VvSuSy*, VIT_11s0016g00470) gene, the sugar transporters *VvTMT1* and *VvHT3* and the sucrose biosynthetic gene *VvSPS* showed increased expression corresponding to increased concentration of ABA. Conversely, *VvSPP*, the downstream sucrose biosynthetic gene to *VvSPS*, showed decreased expression in both ABA and sucrose treatments. The q-PCR results found sucrose phosphate synthase (SPS) gene was consistently up-regulated in response to ABA treatment (**Supplementary Figure 4**), and similarly sucrose transporter (SWEET) was up-regulated by ABA treatment at 2 and 8% sucrose only. Vacuolar invertase (GIN1) was not responsive and the sucrose-6F-phosphate phosphohydrolase (SPP) gene showed no upregulated response to either treatment.

As malic acid content was found to decrease in berries cultured in increased ABA concentration, malic acid related genes were investigated. Fumarate hydratase (*VvFH*) showed reduced expression with increased sucrose concentration and was unresponsive the ABA treatments (**Figure 6C**). The malic acid biosynthetic gene *VvPEPC* showed marked reduction in expression for all treatments with either higher ABA concentration or sucrose concentration while the contrast was observed for the expression of *VvPEPCK* (**Figure 6C**). Different malic enzyme (ME) genes responded differently to changes in ABA and sucrose concentrations. Expression of NADP-ME1 and NADP-ME2 showed an increasing trend with increased sucrose level and ABA level, respectively. NADP-ME3 was upregulated with either higher ABA concentration or sucrose concentration, although the ABA effect was more muted at higher sucrose (**Figure 6C**). The expression of one mitochondrial NAD-dependent ME (NAD-ME) gene was reduced with both ABA and sucrose treatments (**Figure 6C**). No q-PCR analysis of malic acid gene expression was undertaken.

**Figure 7:**
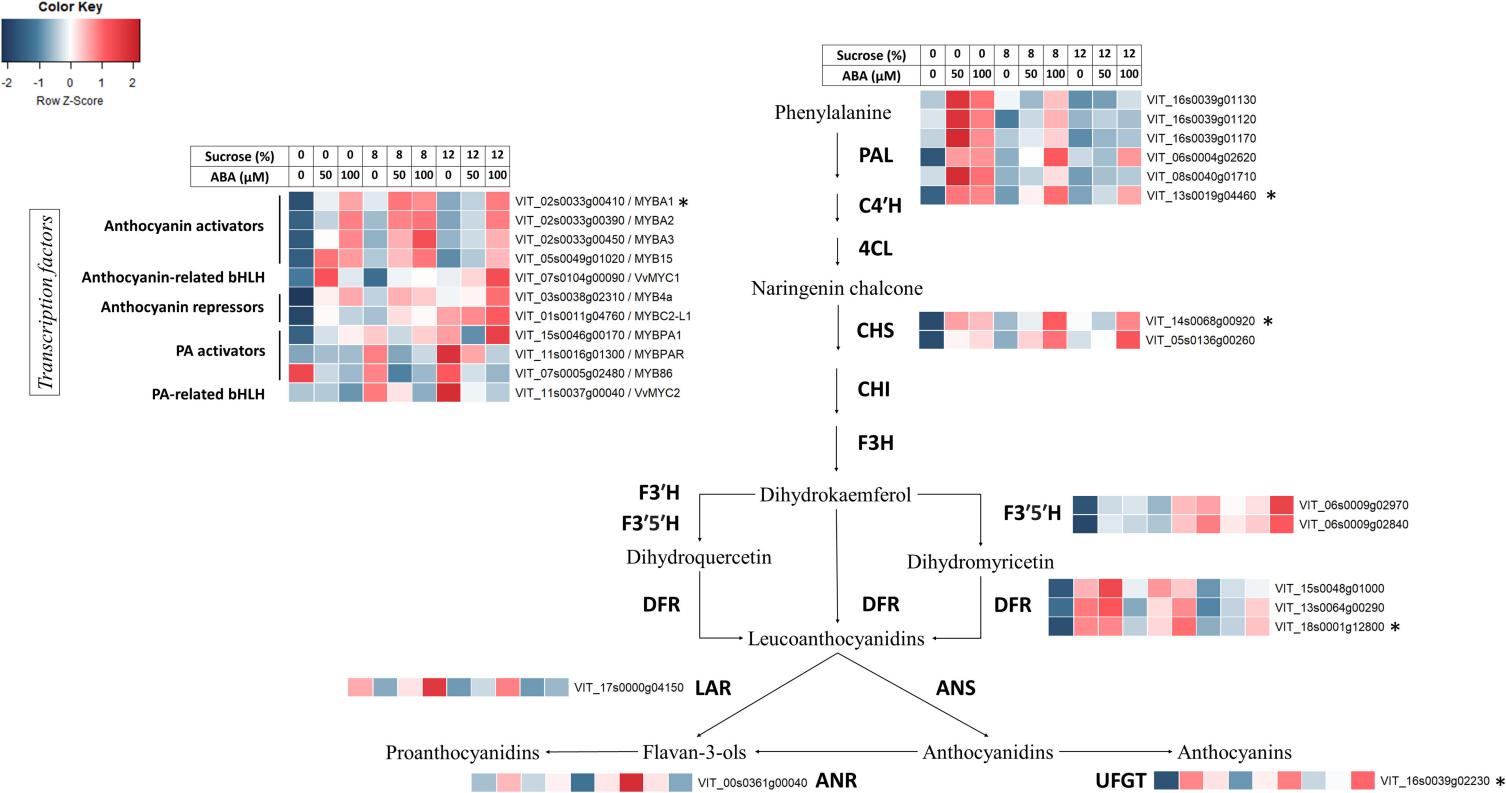
The effect of culture medium sucrose and abscisic acid (ABA) concentration treatment on RNA-seq derived expression of Pinot Noir berry genes from the anthocyanin and proanthocyanidin biosynthesis pathway. Data were Z-score standardised to -2 to 2 for each gene. Genes with FPKM count <2 for all treatments were excluded. *Gene expression also determined by quantitative polymerase chain reaction (qPCR).

**Figure 8:**
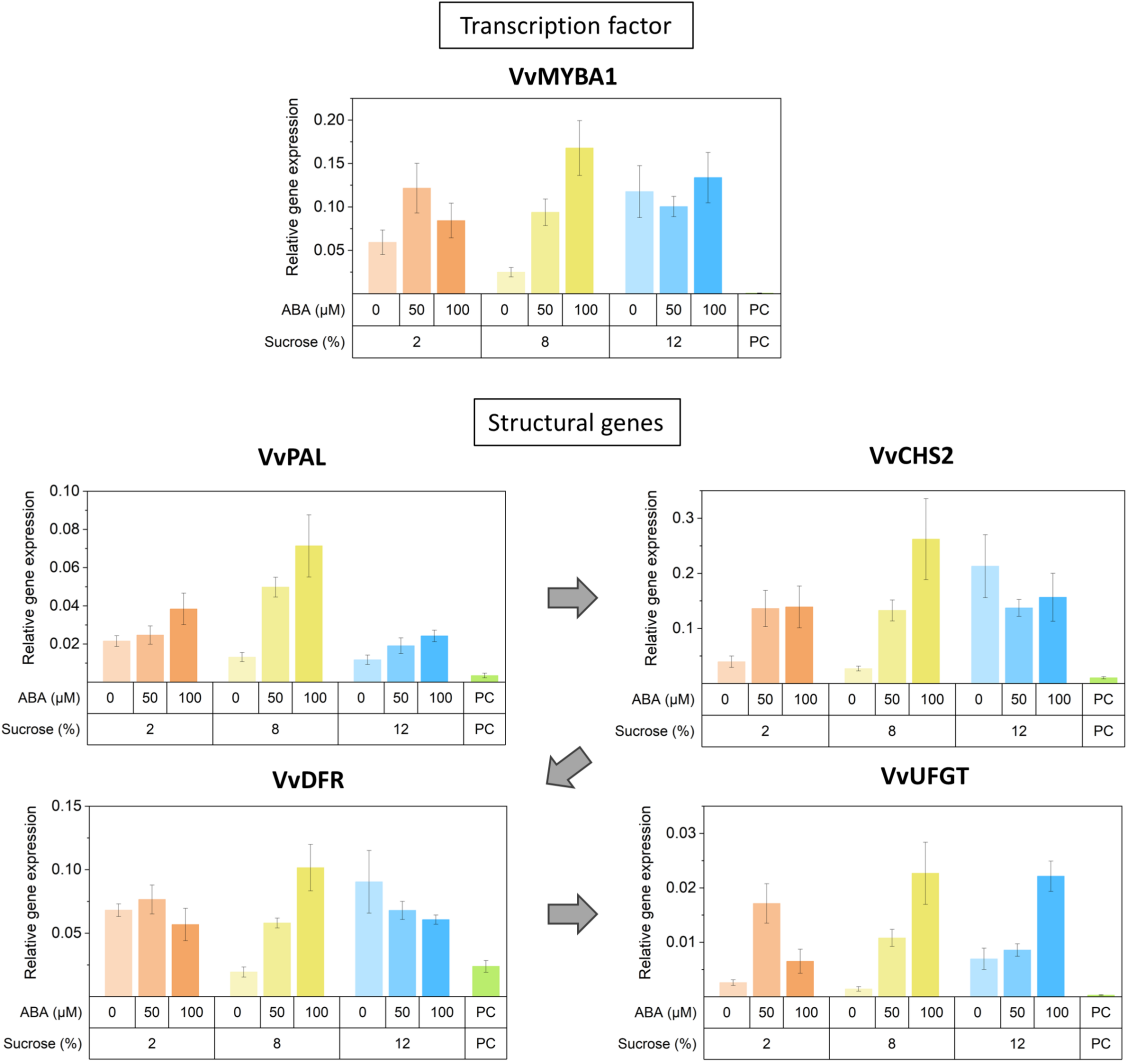
The effects of culture medium sucrose and abscisic acid (ABA) concentration treatments on the expression Pinot noir berry phenylpropanoid pathway structural genes, including phenylalanine ammonia lyase (PAL), chalcone synthase (CHS), dihydroflavonol 4-reductase (DFR), UDP-glucose:flavonoid 3-O-glucosyltransferase (UFGT) and the transcription factors MYBA1 and MYB5A after 15 days of *in vitro* culture. Graphs display average relative expression compared with those of the housekeeping genes GAPDH and Actin. Error bars = standard deviation of at least 3 technical quantitative polymerase chain reaction (qPCR) replicates. PC, pre-culture sample.

Reflecting the observed increase in anthocyanin concentration, transcriptional abundance of anthocyanin-related MYB activators (*VvMYBA1*, *VvMYBA2*, *VvMYBA3* and *VvMYB15*), bHLH TF *VvMYC1* and anthocyanin biosynthetic genes (*VvPAL*, *VvCHS1/2*, *VvDFR*, *VvF3’5’H*, *VvUFGT*) increased with high concentration of ABA/sucrose (**Figure 7**). The expression of these followed a mostly stepwise upward trend following ABA concentration regardless of sucrose treatment. Elevated sucrose concentration alone also resulted in an increase in expression of anthocyanin-related genes though this up-regulation is to a lesser extend compared to the effect of ABA. Expression level of known negative regulators of the general phenylpropanoid pathway such as *VvMYB4A* and *VvMYBC2-L1* also increased with ABA and sucrose concentration. The q-PCR analysis found the expression of PAL, UFGT and CHS2 genes was up-regulated in the presence of ABA, although in the highest sucrose treatment, ABA did not up-regulate CHS2 (**Figure 8**). DFR appeared to be up-regulated in response to ABA at 8% sucrose, but not at either 2 or 12% sucrose. The transcription factor MYBA1 showed a similar response to that of CHS2.

For proanthocyanins, while high ABA and sucrose concentration showed no clear effect on the expression of *VvANR*, there was a reduction in transcript abundance of *VvLAR1* at high ABA concentration (50μM or 100μM) (**Figure 7**). Expression of PA-related transcription factors such as *VvMYBPA1*, *VvMYBPAR*, *VvMYB86* and *VvMYC2* were generally reduced by ABA treatment at any sucrose concentration and their expression pattern was similar to that of *VvLAR1*. Higher sucrose concentration in the absence of ABA generally increased the expression of said transcription factors (**Figure 7**).

## 5 Discussion

### 5.1 ABA and sucrose treatments induced changes in berry ABA and sugar metabolism

The results showed strong evidence for the uptake of exogenous ABA and sucrose into Pinot noir berries during the 15 days of *in vitro* culture. Compared with the pre-culture berries, the total ABA pool of ABA treated berries increased on average by 300%. The concentration of ABA-GE (ABA-storage form), free ABA along with the downstream catabolism metabolites phaseic and dihydrophaseic acids, which act in equilibrium to regulate free ABA within plants (Zeevaart and Creelman, 1988), were all increased significantly (**Figure 3**). RNA seq and qPCR analyses also showed changes to the expression of a number of genes involved in ABA perception (e.g. VvPP2C), signalling (e.g. VvSnRK2F, VvNAC26, VvARM-L), biosynthesis (e.g. VvNCED) and catabolism (e.g. VvCYP7A4) (**Figure 6A**; **Supplementary Figure 3**). Exogenous application of ABA to Cabernet Sauvignon berries on the grapevine was previously found by Wheeler et al. (2009) to increase berry abscisic concentration and expression of two genes implicated ABA synthesis (NCED1 and NCED2). Our findings illustrate cultured Pinot noir grapes also displayed a comprehensive ability to metabolise exogenous ABA resulting in a heightened state of ABA activity within the berry.

In contrast to ABA, the differential uptake of sucrose into berries across the sucrose or ABA treatments was more modest, with only small changes in fructose and total sugar concentration at higher sucrose concentrations (**Table 1**). However, berry sugar content across all berries did increase on average by 31% compared with pre-culture berries, suggesting berries, even on lower sucrose media, had the ability to accumulate sugar during the course of the experiment (**Table 1**). Despite the modest changes in berry sugar concentration, the RNA-seq and qPCR results indicated the berries were being primed by sucrose and ABA treatments. Differential gene expression associated with sugar metabolism was evident, for example, the expression of sugar transporter (*VvSWEET15*) and hexokinases *VvHXK1/2*) was up-regulated by sucrose and ABA (**Figure 6B**; **Supplementary Figure 4**). Consistent with this result was the increased invertase (ß-fructofuranosidase) enzyme activity found by Perez et al. (2000) in Sultana berries cultured on media containing more sucrose (up to 15%). Other sugar transporters (*VvTMT1* and *VvHT3*), sucrose synthase (*VvSuSy*) and other sugar biosynthetic genes (e.g. *VvSPS*) were up-regulated by ABA (**Figure 6B**). In contrast, other downstream sucrose biosynthetic genes (*VvSPP*, *VvSPS* and *GIN1*) showed neutral responses or tended to be down-regulated by sucrose and ABA possibly indicating a feedback response to increased sucrose supply. The complex involvement of berry ABA suggests it has a role in regulation of metabolic sugar genes and therefore potentially berry sugar accumulation in Pinot noir berries. Recently Li et al. (2022) found evidence that sugar accumulation (fructose and glucose) may be stimulated by the phenylpropanoid transcription factor *VvMYB15* in a cascade of *VvGRIP55*-*VvMYB15*-*VvSWEET15* expression in grape berries when ABA activity is increased in the grapevine (root restriction induced ABA). Our RNA-seq data also revealed an up-regulation of *VvMYB15* in berries treated with and exhibiting increased ABA activity (**Figure 6A**). Conversely, there was no evidence that sucrose treatment was influencing regulation of ABA-related genes.

In addition to influence of ABA on the expression of some sugar-related genes, we also found a marked reduction in malic acid concentrations, particularly at lower sucrose, while tartaric acid remained unaffected (**Table 1**). While the positive influence of ABA on grape berry ripening (colour and sugar accumulation) is well understood, there appears to be little understanding of its influence on grape berry organic acids. In sweet cherry the exogenous application of ABA 30 days after full bloom was found to reduce malic acid contents (Kondo and Inoue, 1997), and in tomato Barickman et al. (2016) found that foliar spays or root trickle irrigation application of ABA to pot-grown tomato plants significantly decreased malic acid. In grape, links between ABA and malic acid have also been illustrated. Niculcea et al. (2014). found elevated ABA contents in ripening berries and reduced malic acid concentration in harvest juice (must) from Tempranillo and Graciano grapevines subjected to early-season water deficit regimes. In more recent studies, Wang et al. (2021; 2022) observed that exogenous ABA application to Cabernet Sauvignon bunches accelerated the decrease in malic acid content during berry ripening. Our RNA-seq results suggest that the reduction in malic acid in berries was correlated to numerous changes in the genetic regulation of malic acid biosynthesis and metabolism. Consistent with cessation of malic acid biosynthesis at around véraison (Mullins et al., 1992) was a marked reduction in expression of the malic acid biosynthetic gene (*VvPEPC*) in almost all treatments, while increased expression was found for *VvPEPCK* (**Figure 6C**), a gene associated the dissimilation of malic acid during gluconeogenesis (Kanellis and Roubelakis-Angelakis, 1996). Another biosynthetic gene Fumarate hydratase (FH) which catalyses malate synthesis in the tricarboxylic acid cycle (Sweetman et al., 2009) did not respond to berry abscisic activity. However, the expression of malic enzymes genes (ME) which participate in malate degradation, via catalysing the reversible conversion of malate into pyruvate in the TCA cycle (Ruffner et al., 1984; Sweetman et al., 2009), were correlated with the reduction in malic acid. Cytosolic NADP-dependent ME2 gene expression was increased in berries with higher ABA activity regardless of sucrose treatment, while the expression of NADP-ME1 and NADP-ME2 genes was increased by both sucrose and ABA activity (**Figure 6C**).

### 5.2 ABA and sucrose treatments elicit a cascade of phenylpropanoid pathway gene expression associated anthocyanin synthesis and accumulation

Our results demonstrate sucrose and ABA additions to culture media resulted in modest increase in berry sugars, a significant increase in ABA concentration and activity (gene expression and metabolites) along with a concomitant increase in anthocyanins and berry colour (**Table 1**; **Figures 2**, **4**). While the findings show that sucrose and ABA individually elicit increases in anthocyanin, consistent with previous studies (Hiratsuka et al., 2001; Deis et al., 2011; Dai et al., 2014; Koyama et al., 2010), the combination treatments showed ABA had the ability to increase anthocyanin concentration in low sucrose treatments. For example, in the presence of 2–8% sucrose, the concentration of anthocyanin ranged from 50–100mg/Kg Fwt (**Figure 4**). But, when ABA was applied and the ABA concentration in the berry increased, the anthocyanin concentration rose to around 200mg/Kg Fwt, which is comparable to the concentration observed in berries cultured on high sucrose with or without ABA (**Figures 3**, **4**). Research conducted by Dai et al., 2014 concluded that anthocyanin production in Cabernet Sauvignon berries would only commence once a minimum berry sugar concentration of 72 mg/g Dwt was achieved. Our experiments suggest in general a berry sugar threshold of approximately 30 mg/g Fwt is required to stimulate the anthocyanin accumulation in Pinot noir berries, but if berry abscisic concentration was increased, this was achieved at lower berry sugar concentration. (**Figures 3**, **4**).

In accordance with the changes in the concentration of anthocyanins and other phenolics observed, KEGG and GO analysis of RNA-seq data revealed a broad spectrum of metabolic pathway alteration in response to sucrose and ABA treatments. Key pathways and terms highlighted included: phenylpropanoid metabolism, phenylpropanoid catabolism, flavonoid biosynthesis, phenylpropanoid biosynthesis, plant hormone signal transduction, stilbenoid, diarylheptanoid and gingerol biosynthesis. Along with RNA-seq data the q-PCR results (**Figures 7**, **8**) provide compelling evidence for anthocyanin pathway activation. The effects of both sucrose and ABA are demonstrated for a number of anthocyanin genes (*VvPAL*, *VvCHS1/2*, *VvDFR*, *VvF3’5’H*), but particularly for UFGT, the gene responsible for the final key step in producing pigmented anthocyanins. Here it appeared that ABA was the strongest up-regulator of UFGT at any sucrose concentration. This pattern was also observed for the transcription activator of UFGT, MYBA1 (**Figures 7**, **8**). The transcription of MYB15 has also been implicated to regulate UFGT and F3’5’H in Muscat Hamburg grape berries when vines are exposed to root restriction stress (Li et al., 2021). RNA-seq results found MYB15 expression to be increased in Pinot noir berries treated with and exhibiting increased ABA concentration (**Figure 7**). This finding coupled with the influence of ABA on MYB15 expression in relation to sugar regulation (Li et al., 2022) suggest it may be a pivotal ABA signal transduction mechanism for anthocyanin production in grape.

Interestingly, the cultured Pinot noir berries were found to only accumulate P-3-G and M-3-G (**Figure 5**), the latter being the most abundant anthocyanin found in Pinot noir (Dimitrovska et al., 2011). Consistent with this was the increased expression of F3’5’H gene by sucrose and ABA, particularly so in berries exhibiting increased ABA concentration (**Figure 7**). These observed genetic responses are in agreement with previous transcriptomic studies and ABA experiments by Ban et al. (2003); Jeong et al. (2004) and Koyama et al. (2010). Although it is well established that ABA activates anthocyanin synthesis in both table and wine grapes (Hiratsuka et al., 2001; Gagné et al., 2006; Wheeler et al., 2009; Gambetta et al., 2010; Ferrara et al., 2015; Koyama et al., 2018; Sun et al., 2019), our sugar and ABA *in vitro* culture experiment suggests a predominance of berry ABA over berry sugar in initiating and coordinating the physiological and genetic regulation of anthocyanin synthesis in grape.

Phenylalanine is the key amino acid precursor to the phenylpropanoid pathway (Azuma, 2018) and its accumulation in the grape berry is considered pivotal to the pre-ripening accumulation of phenolics such as proanthocyanins and tannins and the post-véraison accumulation of anthocyanins and flavonols (Mullins et al., 1992). Interestingly, we observed a sucrose effect on phenylalanine with a significant drop in concentration at higher sucrose irrespective of ABA. At low sugar increased ABA concentration was also associated with a reduction in phenylalanine concentration (**Figure 4**). The reduction in phenylalanine appeared to correspond to the overall increase in anthocyanins (**Figure 4**). Similar observations were made by Dai et al. (2014) where Cabernet Sauvignon berries cultured on high sugar media had lower concentrations of phenylalanine but exhibited much higher anthocyanin concentrations. And indeed, changes in the flux between phenylalanine and anthocyanins does correlate with increased PAL gene expression found in treated berries along with previously mentioned increases in CHS and UFGT gene expression (**Figures 7**, **8**).

However, our results also show that in other parts of phenylpropanoid pathway, ABA treatment acted to significantly reduce the hydroxycinnamic acid esters caftaric acid and coutaric acid and the proanthocyanidin catechin regardless of dosage rate, while sucrose slightly reduced total tannins (**Supplementary Figures 2**, **4**). These results are consistent with RNA-seq data which highlighted a sharp down-regulation of the transcription factors MYB86 and MYC2 and reduced expression of the LAR gene (**Figure 7**). While there was no consistent down-regulation of ANR in treated berries (**Figure 7**), previous work by Lacampagne et al. (2010) has illustrated reduced expression of both *VvANR* (anthocyanidin reductase) and *VvLAR1* (leucoanthocyanidin reductase) in Cabernet Sauvignon berries sprayed with ABA solution pre-véraison.

The contrasting up-regulation of anthocyanin genes versus the down-regulation of proanthocyanidin genes found in berries exhibiting increased ABA activity illustrate the complex influence of ABA on the phenylpropanoid pathway in grape. These findings together with the established regulatory action of ABA on other aspects of grape ripening (Fasoli et al., 2018; Tornielli et al., 2023) confirm its pivotal role in the overall coordination of grape berry ripening and maturity.

## 6 Conclusions

The *in vitro* berry culture system successfully illustrated the dual effect of sucrose and ABA on enhancing the ripening of Pinot noir berries, in particular the increased colour development and corresponding increase in anthocyanins. This finding, along with the other results presented, illustrate the utility of the berry culture system and provide alternative experimental approaches to traditional field trials to investigate the effects of nutrients and hormones on berry ripening. Indeed. we believe this approach has scope in the investigation of many other aspects of berry ripening, including the regulation of grape derived wine flavours and aromas and in the general understanding of berry physiology, biochemistry and the underpinning genetic regulation.

Exogenous ABA supply and increased ABA concentration in the berry had the ability to boost anthocyanin production in berries when sucrose supply was low. Much of ABA’s stimulatory effect on berry anthocyanins was found to be manifested in a cascade of up-regulated transcription factors and genes in the phenylpropanoid pathway, while in other parts of the pathway a down-regulation of key proanthocyanidin transcription factors and biosynthetic genes corresponded to a reduction in berry proanthocyanidins irrespective of sucrose supply. Similarly, increased ABA concentration was correlated with a significant reduction of berry malic acid concentration. Together these results suggest a predominance of berry ABA over berry sugar in coordinating the physiological and genetic regulation of anthocyanins and proanthocyanins and indeed grape ripening in general. By determining the individual and combined effects of ABA and sugar, our findings contribute to a better understanding of the underlying mechanisms governing berry ripening and provide insights into how the physiology of ABA in berries could be manipulated to enhance the anthocyanin based colouration of grape berries, particularly when berry sugar supply is low.

## Data availability statement

The original contributions presented in the study are included in the article/**Supplementary Material**. Further inquiries can be directed to the corresponding author.

## Author contributions

JB: Data curation, Formal analysis, Investigation, Methodology, Supervision, Writing – original draft, Writing – review & editing. SM: Data curation, Investigation, Methodology, Resources, Validation, Writing – review & editing. HN: Data curation, Formal analysis, Software, Writing – review & editing. HB: Data curation, Formal analysis, Investigation, Writing – review & editing. JC: Data curation, Formal analysis, Investigation, Methodology, Writing – original draft, Writing – review & editing. CE: Data curation, Formal analysis, Writing – review & editing. LA: Data curation, Formal analysis, Writing – review & editing. PB: Data curation, Methodology, Supervision, Writing – review & editing. KL-W: Data curation, Formal analysis, Methodology, Software, Writing – review & editing. BP: Data curation, Formal analysis, Investigation, Writing – review & editing. DM: Funding acquisition, Writing – review & editing. RE: Project administration, Writing – review & editing.
