# Supplementary material for: Exogenous abscisic acid and sugar induce a cascade of ripening events associated with anthocyanin accumulation in cultured Pinot Noir grape berries
Source: Front Plant Sci. 2023 Dec 21;14:1324675. doi: 10.3389/fpls.2023.1324675 (PMC10768192; doi:10.3389/fpls.2023.1324675)
Supplement: Supplementary file 1 [file Image_1.pdf]

Supplementary Figures

A)

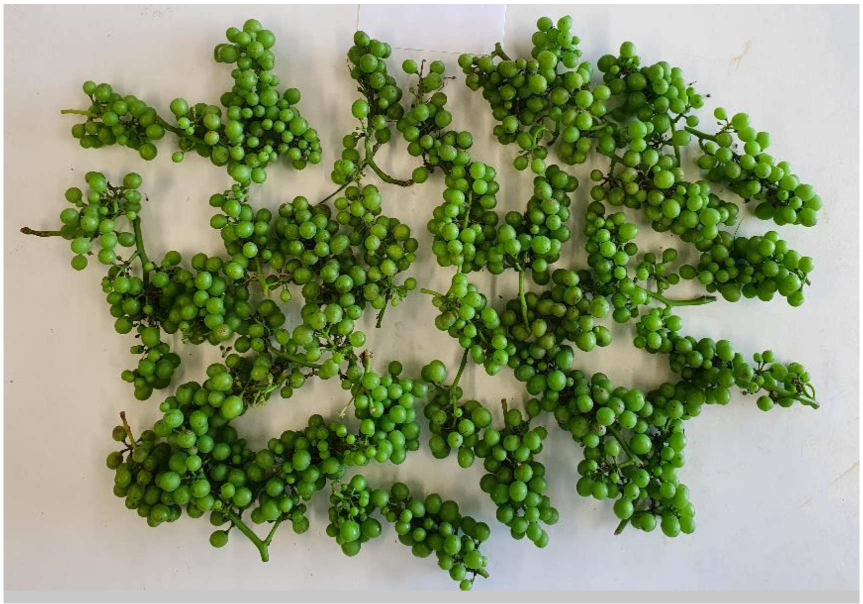

B)

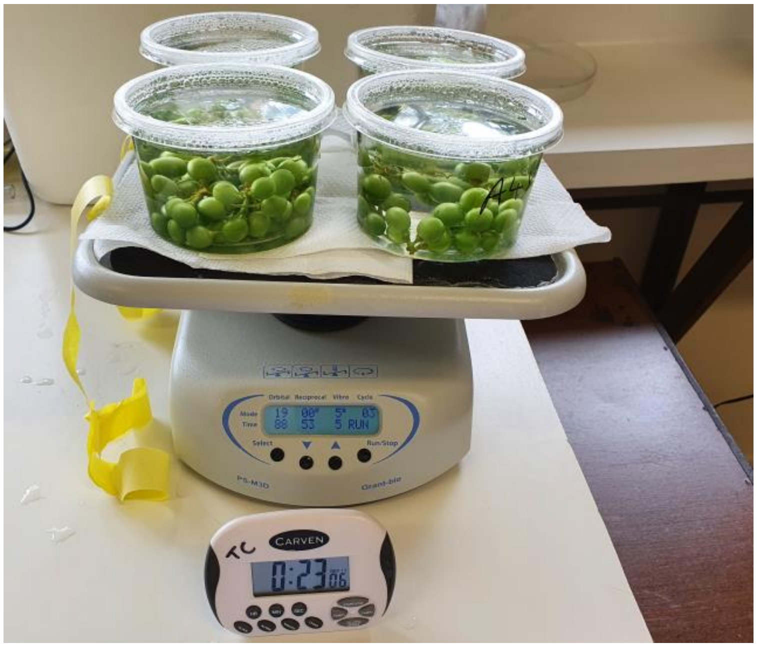

C)

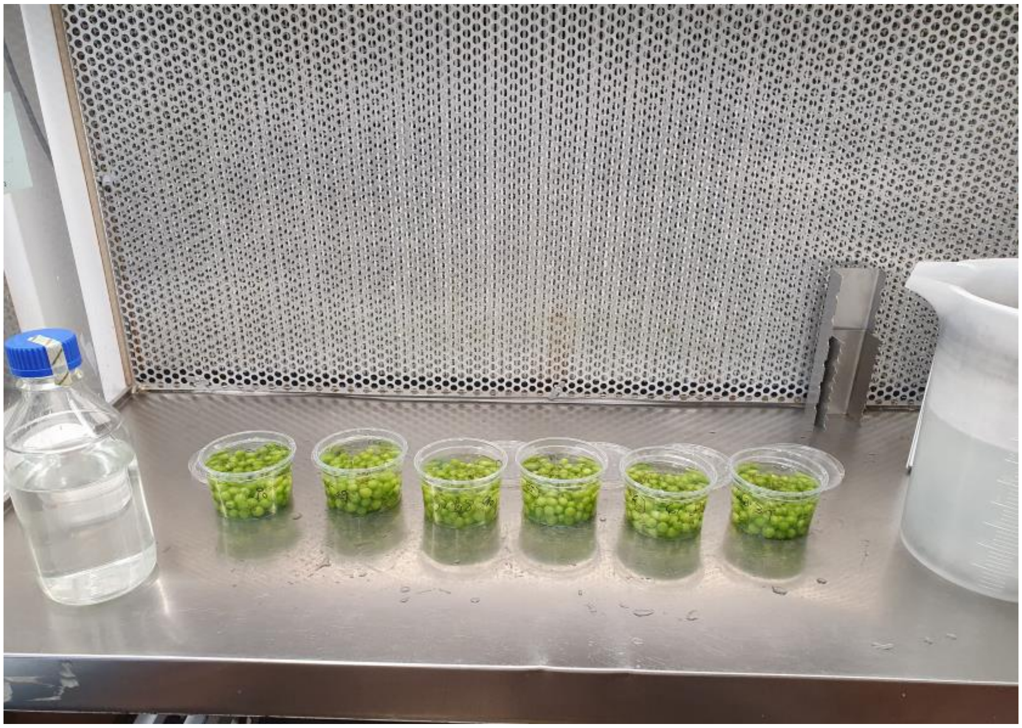

D)

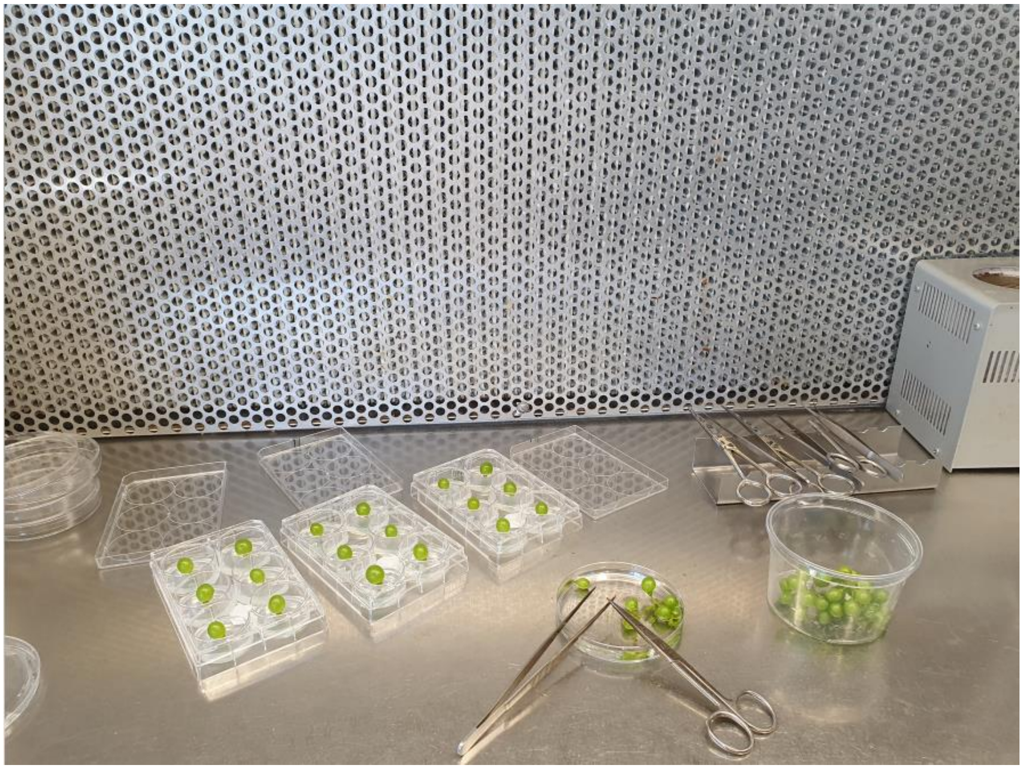

E)

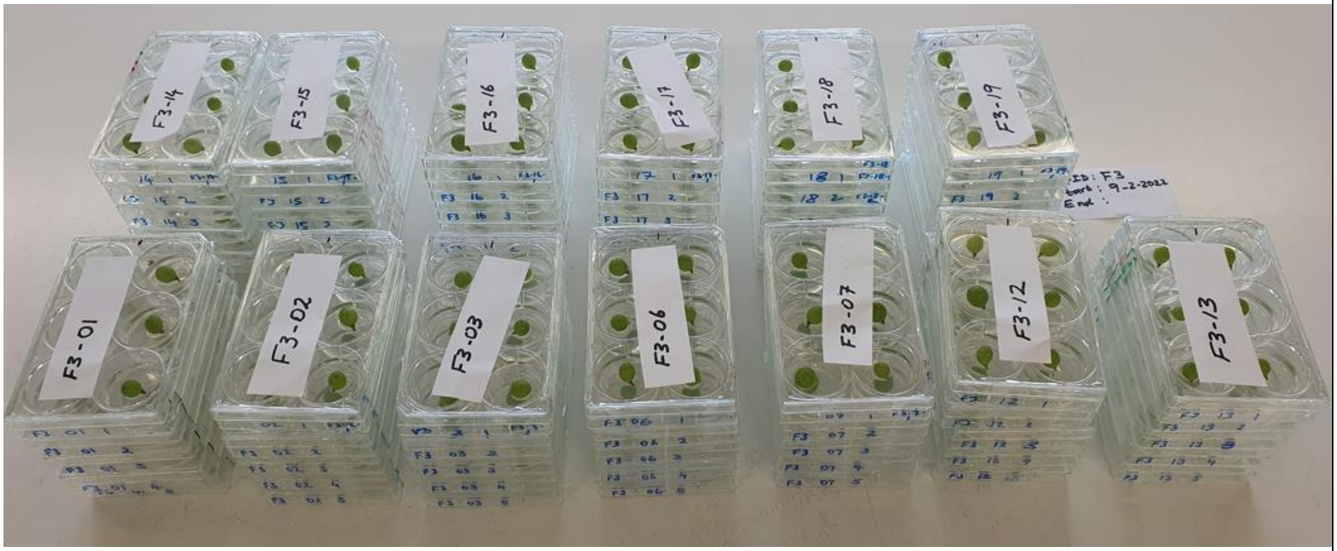

Supplementary Figure 1: Pinot noir berry culture procedure.

A) Field-grown grape berries were pooled together for experimentation.

B) Pre-culture sterilisation of berries involved immersion in a 0.5% (w/v) sodium dichloroisocyanurate solution with two to three drops of Tween® 20 detergent for 25 minutes, using continuous gentle agitation.

C) Following sterilisation, berries underwent a triple rinse with sterile distilled water within a laminar flow hood.

D) Surface-sterilised berries were aseptically treated with a sterile 20 mM EDTA solution. Pedicels were trimmed to approximately 3 mm within the same EDTA solution before placement in respective 6-well culture plates.

E) Green pre-véraison Pinot noir berries, prepared and ready for in vitro berry culture incubation at 24°C.

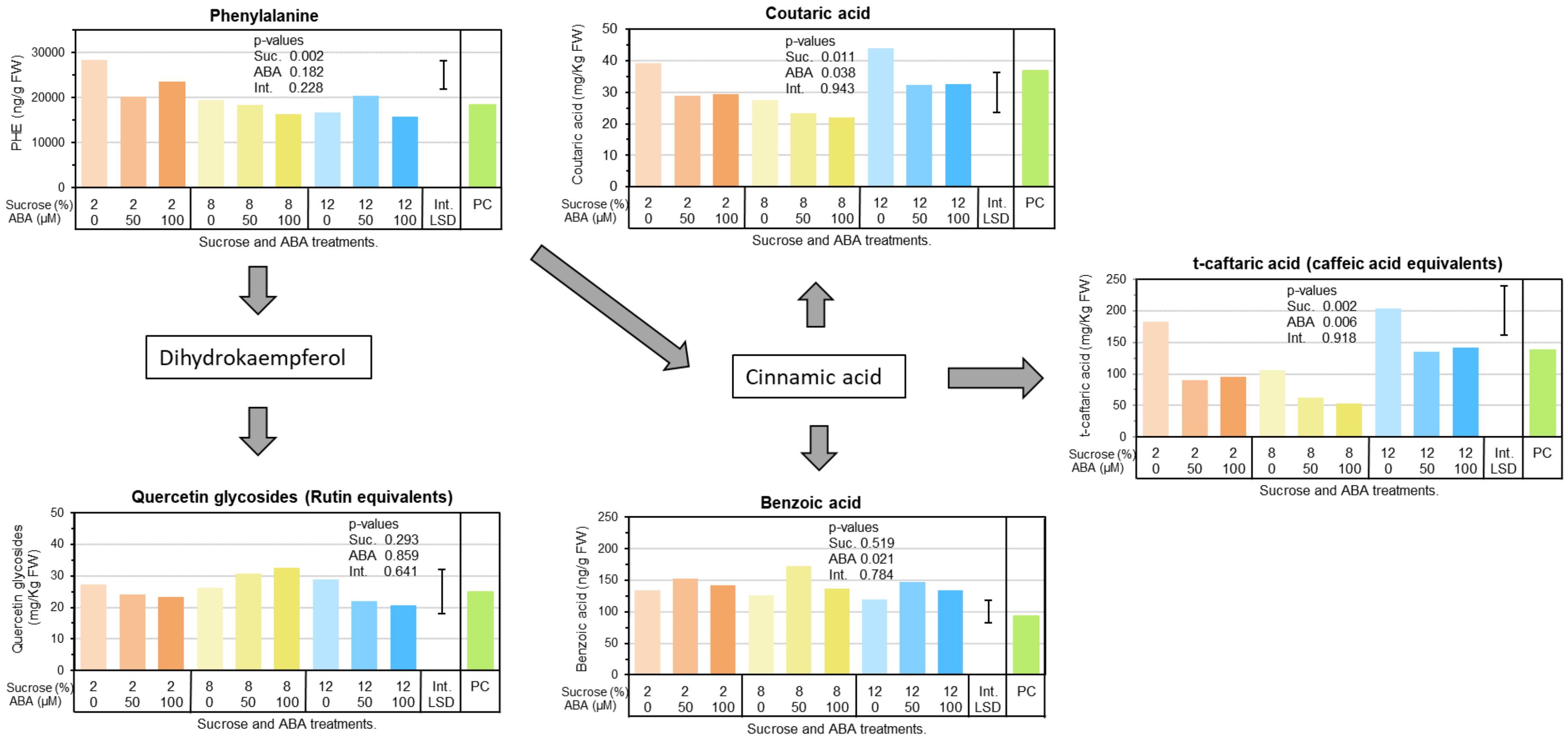

Supplementary Figure 2. The effects of culture medium sucrose and abscisic acid (ABA) concentration treatments on Pinot noir berry phenylpropanoid pathway metabolism, including berry concentrations of phenylalanine, phenolic acids and quercetin glycosides after 15 days of *in vitro* culture. Note pre-culture (PC) is not statistically compared with sucrose and ABA treatments. P-values for main and interaction effects are presented in each graph. Error bars represents interaction LSD (α = 5%).

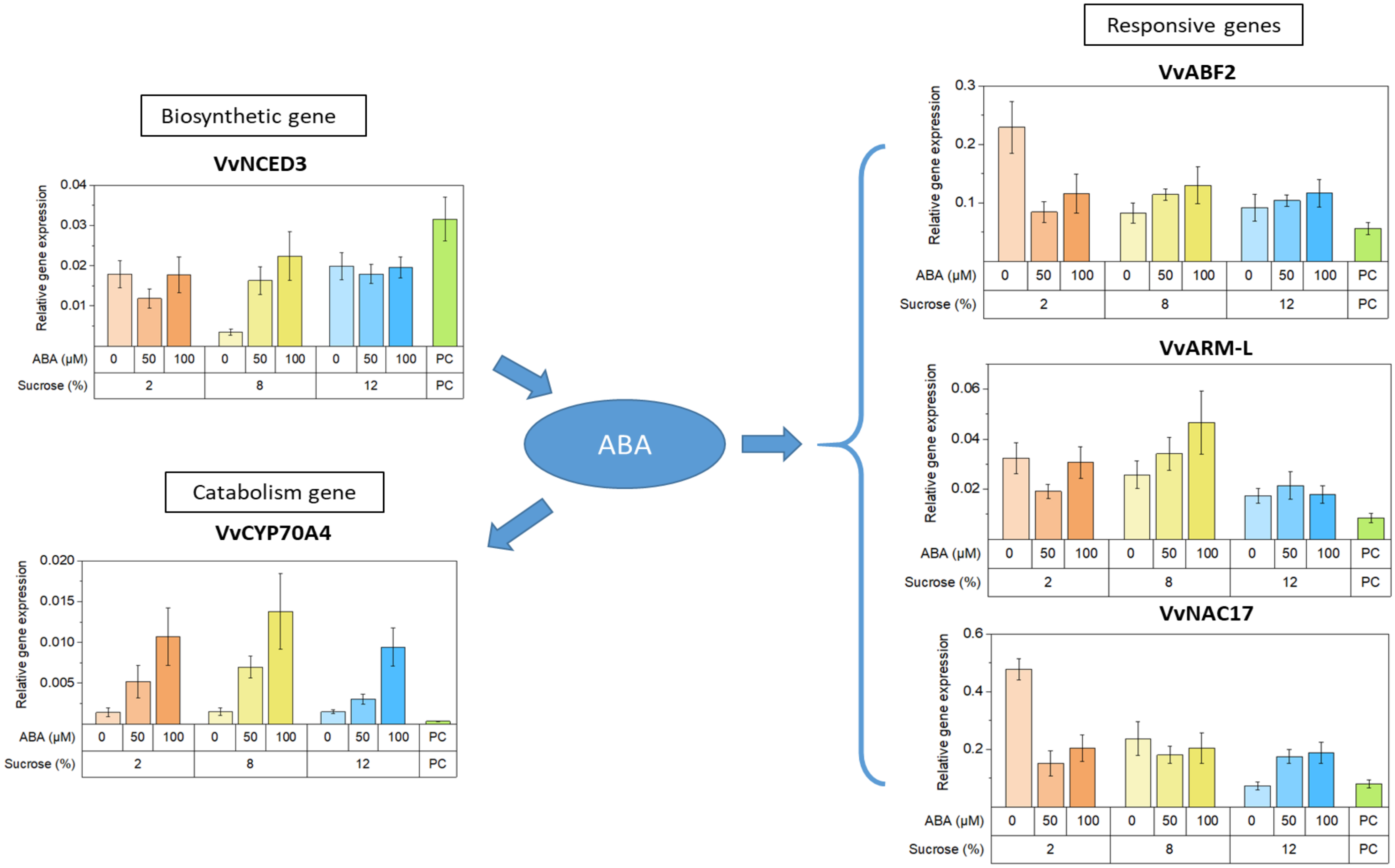

Supplementary Figure 3. The effects of culture medium sucrose and abscisic acid (ABA) concentration treatments on the expression of Pinot noir berry abscisic acid responsive and catabolism genes, including nine-cis-epoxycarotenoid dioxygenase 3 (NCED3), abscisic acid 8'-hydroxylase 4 (CYP707A4), abscisic acid responsive elements-binding factor 2 (ABF2), armadillo-like (ARM-L), NAC-domain protein 17 (NAC17) after 15 days of *in vitro* culture. Graphs display average relative expression compared with those of the housekeeping genes GAPDH and Actin. Error bars = standard deviation of at least 3 technical quantitative polymerase chain reaction (qPCR) replicates. PC = pre-culture sample.

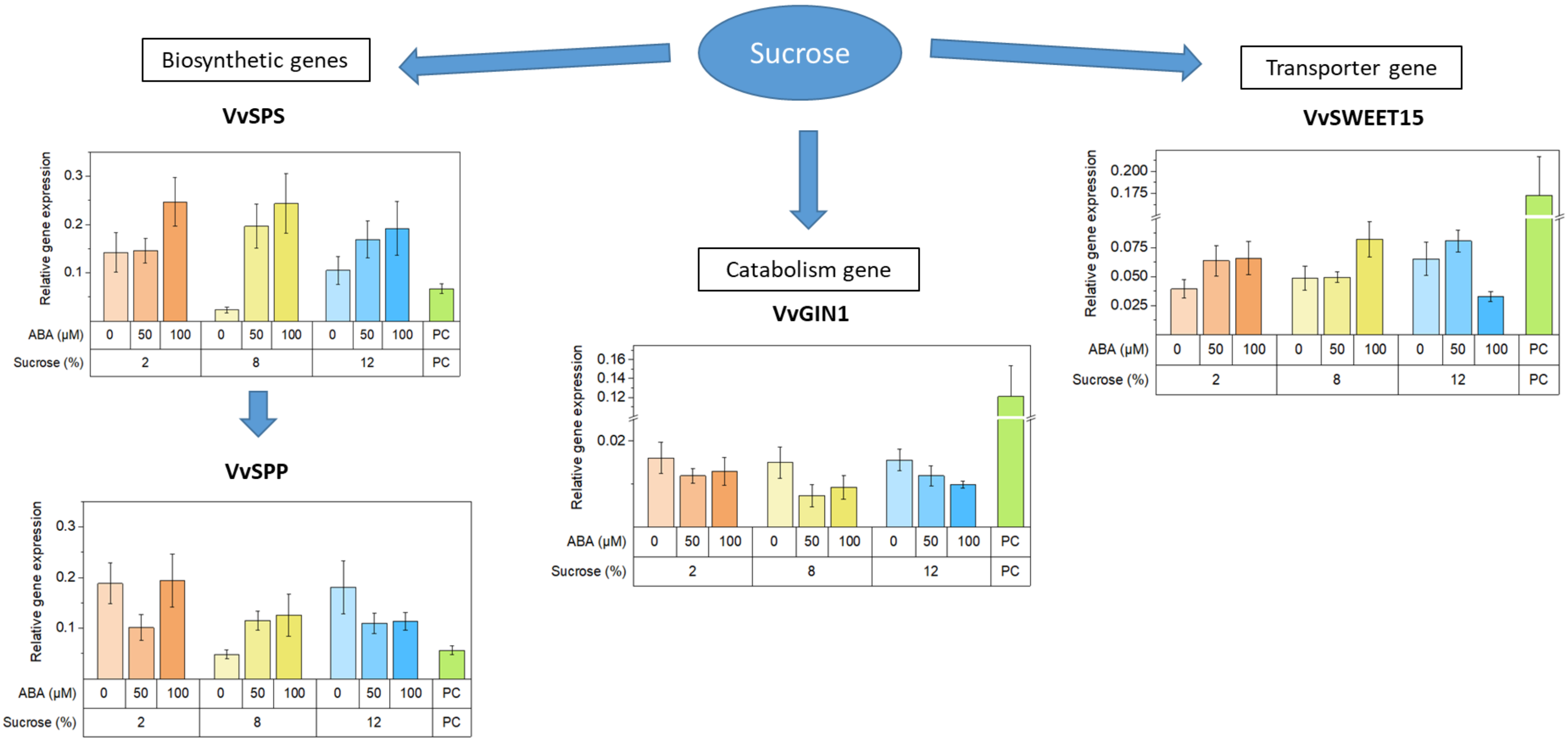

Supplementary Figure 4. The effects of culture medium sucrose and abscisic acid (ABA) concentration treatments on the expression of Pinot noir berry sugar synthesis, transporter and metabolism genes, including sucrose-6F-phosphate phosphohydrolase (SPP), sucrose phosphate synthase (SPS), vacuolar invertase 1 (GIN1), sucrose transporter (SWEET15). Graphs display average relative expression compared with those of the housekeeping genes GAPDH and Actin. Error bars = standard deviation of at least 3 technical quantitative polymerase chain reaction (qPCR) replicates. PC = pre-culture sample.

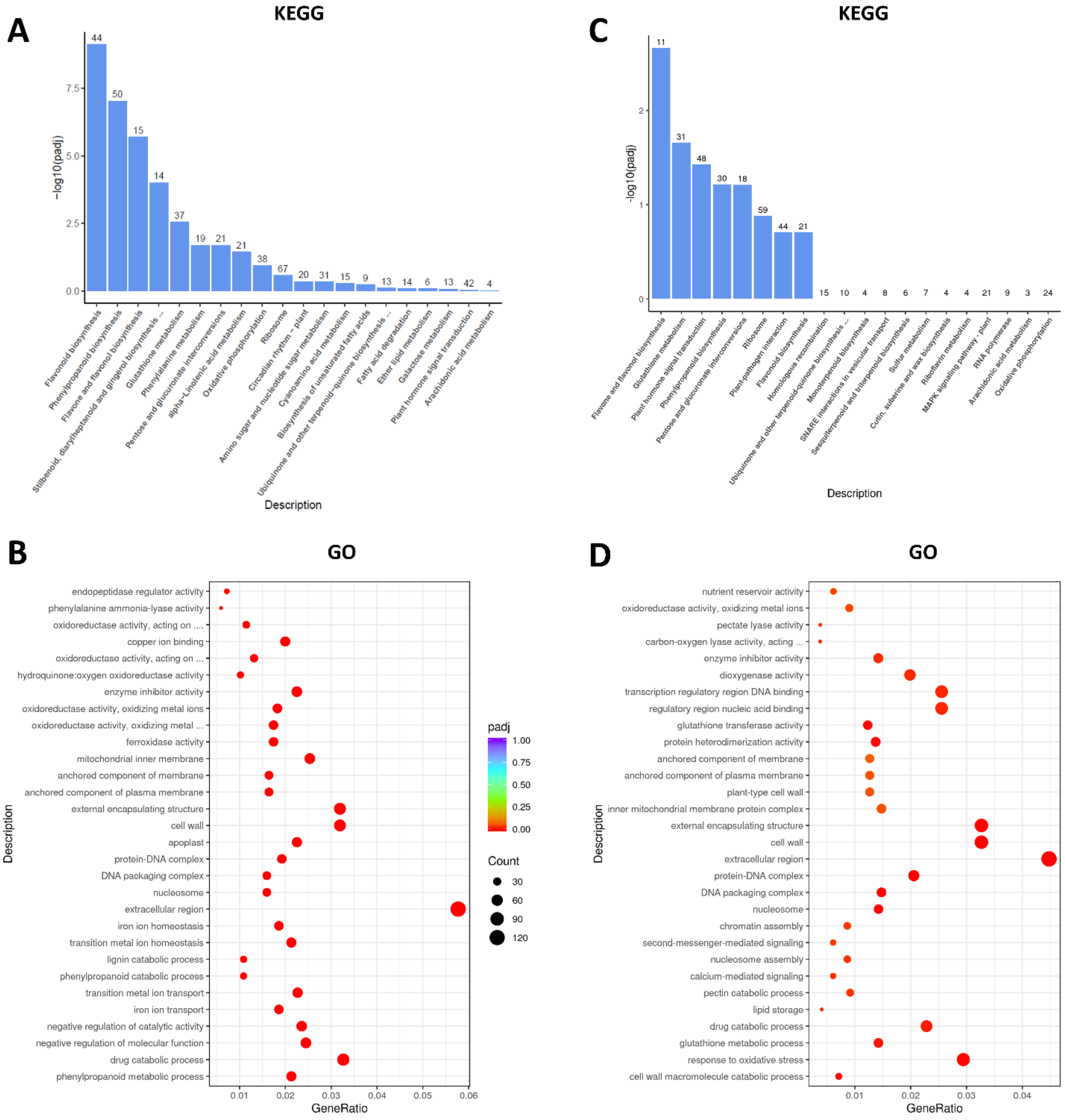

Supplementary Figure 5. GO and KEGG pathway enrichment analysis of upregulated DEGs. (A & B) At 2% medium sucrose, 0µM ABA vs 50 µM ABA. (C & D) At 0µM ABA, 2% sucrose vs 8% sucrose. For GO enrichment analysis, the 30 most significant GO terms are displayed. GeneRatio is the ratio of DEGs to all genes within each GO term. For KEGG pathway enrichment analysis, the 20 most significant pathways are displayed. Annotated number for each KEGG pathway represents the number of DEGs in the pathway.
