# Supplementary material for: Exogenous abscisic acid and sugar induce a cascade of ripening events associated with anthocyanin accumulation in cultured Pinot Noir grape berries
Source: Front Plant Sci. 2023 Dec 21;14:1324675. doi: 10.3389/fpls.2023.1324675 (PMC10768192; doi:10.3389/fpls.2023.1324675)
Supplement: Supplementary file 2 [file Table_1.docx]

Supplementary Tables

Supplementary Table 1. Sequences of the DNA primers used in quantitative polymerase chain reaction (qPCR) analysis.

| Gene | Forward Primer Sequence | Reverse Primer Sequence |
| --- | --- | --- |
| PAL | CGGAGGAGGCGAGAGAGGGAGTA | GTGGCAAGATTCCGTCCCGTTT |
| CHS | GAAGATGGGAATGGCTGCTG | AAGGCACAGGGACACAAAAG |
| DFR | CACATGAGAAACCTGTAGATGGCAAGA | GCCAAATCAAACTACCAGAAAACCTTG |
| UFGT | GAGCTTTAGGCAGGGGATGGTGAT | AGTCCAAAACGGCAGCCAAGC |
| MYBA1 | TGGCATAGTCACCACTTCAAAAAGG | GGATGATGGCTTCCTGGAAGTA-CTG |
| SPP | AGAATGGCAGTGTTGCATGACC | TGATCTTTAGCACCTGAGCCTTCC |
| SPS | ATAATGGCGTCTCGTTCCCAAG | CCCCACCAAGCAATCCTTCATA |
| GIN1 | TCTTTGGCCGTGGATGATGTTG | CCCCTTGGCTAAAGCCTTCCAC |
| SWEET15 | TGGTCATGCTGAGCACATTAGGC | TCTCGCGATTCCCAGTTTCTTCA |
| NCED3 | CCTCTCAACTGGGCAAGTCAGC | GCACAAAAGCCAGGATGTACCC |
| CYP707A4 | TTCCTGACCCTCAGAAGTTTGATCC | GGTGGTGGGTCATAATAAGCATCTCC |
| ABF2 | TGGAATTGGAAGCAGAGGTTGC | GCCAGTCAGTGTGCGTCTCAAG |
| ARM-L | TTTGCTGTCTCTTTGCATCAATGG | TTATGTATGATTTTGAGGAGCGAGCAG |
| NAC17 | CAAGCCGAAGAAGAAGTCCAGAGC | TGCCTATATCCAAATCCACTAGGTTGG |
| Actin | GCCCCTCGTCTGTGACAATGGA | CCAACCATGACACCAGTGTGCC |
| GAPDH | GCUGCCAUCAAGGAGGAGUCAGA | TCTCGTTGAGTGCTATTCCAGCCTT |
